# Preoperative imatinib and laparoscopic intersphincteric resection for large rectal gastrointestinal stromal tumor: A case report

**DOI:** 10.1016/j.ijscr.2020.05.031

**Published:** 2020-05-23

**Authors:** Shinnosuke Nagano, Norikatsu Miyoshi, Tsuyoshi Takahashi, Hiroaki Itakura, Shiki Fujino, Takayuki Ogino, Hidekazu Takahashi, Mamoru Uemura, Chu Matsuda, Tsunekazu Mizusima, Masaki Mori, Yuichiro Doki

**Affiliations:** a Department of Gastroenterological Surgery, Osaka University Graduate School of Medicine, 2-2-E2 Yamadaoka, Suita City, Osaka 565-0871, Japan; b Department of Innovatice Oncology Research and Regenerative Medicine, Osaka International Cancer Institute, 3-1-69 Ohtemae, Chuo-ku, Osaka 541-8567, Japan; c Department of Surgery and Science, Kyusyu University Graduate School of Medicine, 3-1-1 Maidashi, Higashi-ku, Fukuoka City, Fukuoka 821-8582, Japan

**Keywords:** GIST, gastrointestinal stromal tumor, IM, imatinib mesylate, ISR, intersphincteric resection, Rectal gastrointestinal stromal tumor, Imatinib, Laparoscopic intersphincteric resection

## Abstract

**Introduction:**

Anus-preserving surgery for a large rectal gastrointestinal stromal tumor (GIST) may be difficult because of the location of the tumor in the pelvis. Therefore, rectal GIST might require extensive surgery, such as abdominoperineal resection. In recent years, preoperative imatinib therapy has been used to reduce tumor size and preserve the anus in some cases. However, there have been few reports of laparoscopic anal-preserving surgery for giant rectal GIST.

**Presentation of a case:**

We present the case of a 55-year-old man who was referred to our hospital for examination of a 10-cm pelvic mass in the lower rectum. Endoscopic ultrasound with fine needle aspiration was performed, and the pathological findings resulted in a diagnosis of GIST. The mass had spread to the prostate and left levator ani muscles, and as a result, surgery was deemed difficult to perform without damaging the pseudo-capsule. Therefore, preoperative chemotherapy with imatinib mesylate (IM) was performed for 8 months. The mass was reduced to 7.8 cm, and laparoscopic intersphincteric resection (ISR) was performed.

**Discussion:**

We also review prior cases of rectal GIST where patients had undergone anal-preserving surgery following preoperative chemotherapy with IM. Our case represented the largest tumor size in a review of cases of patients who had successful anal-preserving laparoscopic surgery following preoperative chemotherapy with IM.

**Conclusion:**

Preoperative chemotherapy with imatinib mesylate was effective for reducing the rectal GIST, and laparoscopic ISR was useful for anal preservation, even when a tumor is large.

## Highlights

• Rectal GIST is a rare disease, and have often grown by the time of discovery due to lack of initially subjective symptoms.
• A large rectal GIST often require extensive surgery which may lead to postoperative bowel dysfunction.
• Preoperative chemotherapy with imatinib mesylate is effective for reducing the rectal GIST.
• Laparoscopic intersphincteric resection may be a useful technique for giant rectal GIST, which enables anal preservation.

## 1 Introduction

Surgical resection is the preferred treatment for gastrointestinal stromal tumors (GISTs). Due to lack of initially subjective symptoms, rectal GISTs have often grown by the time of discovery, and therefore, anus preservation during surgery is often difficult [1]. Recently, the effectiveness of preoperative chemotherapy with imatinib mesylate (IM) has been shown, resulting in some cases where anal-preserving surgery was possible after tumor reduction. However, there have been few reports of laparoscopic anal-preserving surgery for giant rectal GIST. We present the case of a patient with a 10 cm rectal GIST who was successfully treated with IM and laparoscopic intersphincteric resection (ISR) to preserve the anus. We also review prior cases of rectal GIST where patients had undergone anal-preserving surgery following preoperative chemotherapy with IM. Our case represented the largest tumor size in that review witch was operated laparoscopically.

The work has been reported in line with the SCARE criteria [2].

## 2 Presentation of case

**Fig. 1:**
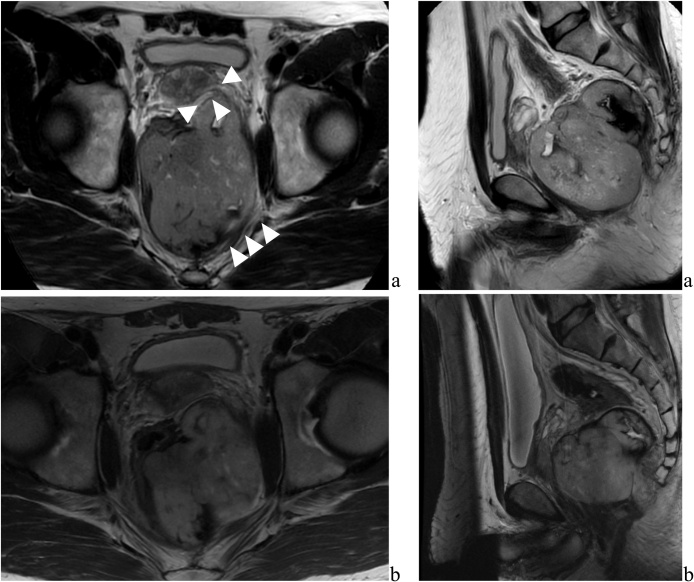
a: Magnetic resonance imaging showed a mass measuring 10 cm in maximum on the left side of the lower rectum. The tumor extended to the left side of the prostate and part of the left levator ani muscle. b: After 8 months preoperative chemotherapy with imatinib mesylate, it showed that marked shrinkage of tumor to 7.8 cm.

A 55-year-old man, with a history of abnormal bowel movements, visited local clinics over a 5-month period. Lower gastrointestinal endoscopy showed no abnormality in the rectal mucosa. An abdominal computed tomography (CT) found a huge mass in the Ra (rectum/above the peritoneal reflection) -Rb (rectum/below the peritoneal reflection) section of the rectum, and the patient was referred to the hospital for thorough examination. Pelvic magnetic resonance imaging (MRI) showed a mass measuring 10 cm on the left side of the lower rectum (Fig. 1). The tumor extended to the left side of the prostate and the left levator ani muscle. Endoscopic ultrasound-fine needle aspiration revealed bundles of spindle cells with positive immunohistochemical staining for c-Kit and DOG-1 but negative for S-100 protein, leading to a diagnosis of rectal GIST. Since it would be difficult to excise the tumor without pseudo-capsule damage, neoadjuvant imatinib (400 mg/day) was initiated. In a CT scan 2 months later, the tumor diameter had shrunk to 9.5 cm and the contrast effects inside the tumor had disappeared (Fig. 1). Eight months after chemotherapy was initiated, the tumor showed marked shrinkage to 7.8 cm, an almost maximum tumor response. It was decided that surgical treatment was possible at this point.

**Fig. 2:**
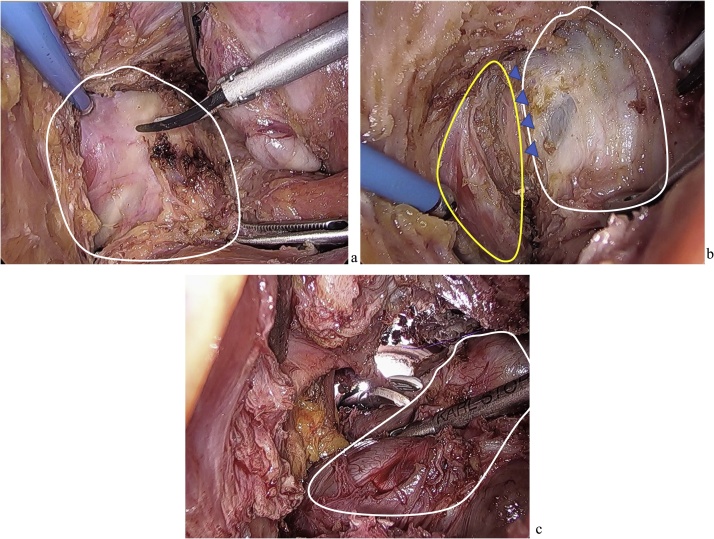
Intraoperative laparoscopic view. a: Large tumor where is left side of the rectum from the view of Intraperitoneal (white line). Without damaging the pseudo-capsule, we dissected the tumor. b: Tumor extending to the left levator ani muscle from the view of Intraperitoneal (yellow line). A part of tumor was attached to it (blue arrow). c: The abdominal cavity and anal side was opened all around by anal-side approach. The white line points to the tumor.

The patient indicated a strong desire to preserve the anus, and therefore, we chose laparoscopic ISR and temporary transverse colostomy (Fig. 2). Surgery was started from mobilizing the descending colon and sigmoid colon. The origin of the inferior mesenteric artery was exposed and dissected. Next, mesorectal excision was performed as much as possible. Around the left side of the rectum, we dissected the tumor without damaging the pseudo-capsule (Fig. 2a). When the tumor location was confirmed by intraoperative rectal examination, we judged that it was difficult to dissect the rectum from the intraperitoneal with a enough anal margin (Fig. 2b), so got started anal-side approach under the microscope. A transanal access platform (GelPOINT Path; Applied Medical) and AirSeal (CONMED) platform was introduced. Mucosal dissection was performed at the height of dentate line. The tumor was extended to the left levator ani muscle, so the mass was dissected with some muscles attached to it. The abdominal cavity and anal side was opened all around (Fig. 2c). The anal side of rectum and oral side of colon was resected, and we removed the specimen. The rectosigmoid colon and anal canal was anastomosed by handsewn suture and a temporary transverse colostomy was performed. The operation time was 608 min, and the blood loss was 130 ml.

**Fig. 3:**
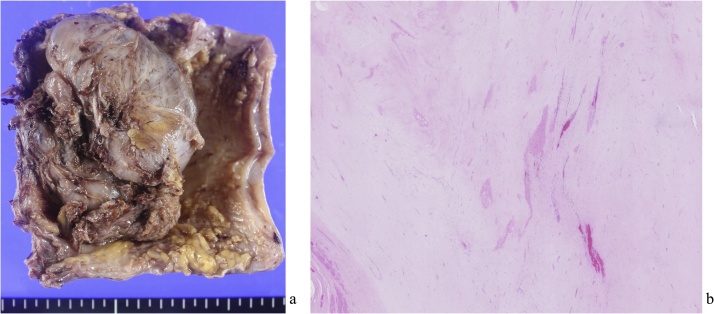
Pathological examinations. a: The resected tumor was 70 × 50 mm in size with necrotic change. b: Histological examination (hematoxylin and eosin staining). Most of the tumor was disappeared by vitrification and fibrosis.

The patient recovered with no postoperative complications and left the hospital on postoperative day 10. In histopathological tissue, most of the tumor disappeared by vitrification and fibrosis (Fig. 3). One year after postoperative chemotherapy with IM, the patient exhibits no evidence of recurrence.

## 3 Discussion

Primary rectal GIST is a relatively rare disease, accounting for approximately 5% of all GIST [3]. The current report presents the case of a patient who had a large rectal GIST that had progressed to other organs and was successfully treated with preoperative IM and anus-preserving anus ISR [1]. The primary treatment for a rectal GIST is complete surgical resection with negative microscopic margins, but this procedure is challenging for surgeons because of the confined pelvic space and the nature of the GIST to densely adhere to the pelvic floor [4]. Therefore, rectal GIST might require extensive surgery, such as abdominoperineal resection or pelvic exenteration, which may lead to postoperative bowel dysfunction [3]. Recently, there have been some reported cases where anal preservation surgery was possible for large tumors following preoperative chemotherapy with IM [1,[5], [6], [7], [8], [9]].

Preoperative chemotherapy with IM is useful for the treatment of rectal GIST. Cvanar et al. studied 83 patients with rectal GIST who underwent preoperative chemotherapy and found a high tumor shrinkage rate. Sixty patients (72.3%) had a partial response (PR), 18 (21.7%) had stable disease (SD), 3 (4.8%) had a complete response (CR), and 1 (1.2%) had progressive disease (PD) according to RECIST guidelines [5]. In addition, in their retrospective series examining surgical margins, 46 of 51 patients (90%) who underwent neoadjuvant Imatinib and surgery had negative margins compared to 33 of 63 (52%) patients who did not [5]. Jakob et al. reviewed 39 patients and reported that preoperative chemotherapy led to improved surgical margins and increased local disease-free and overall survivals [6]. Wilkinson et al. examined 19 patients who underwent extended surgery for rectal GIST and reported that reducing the tumor size with preoperative chemotherapy resulted in an increased likelihood of sphincter-sparing surgery [7]. Thus, preoperative chemotherapy with IM for rectal GIST can be expected to have a tumor shrinking effect, ensure surgical margins that avoid false capsule damage, and improve postoperative oncological outcomes and anal function.

A goal of surgery for GIST is to ensure a surgically safe margin without pseudo-capsule damage [8]. For small tumors, local excision, such as transanal, transsacral, and transperineal approaches, may be selected since they are minimally invasive and preserve defecation function. On the other hand, local excision may experience a poor surgical field of view, and therefore, it may be difficult to ensure the surgical margin. Jakob et al. examined 36 patients who underwent surgical treatment for rectal GIST and reported that all cases with local recurrence had undergone local excision with positive margins and with no preoperative Imatinib [6]. They concluded that local resection should only be performed when microscopically clear margins can be safely achieved [6].

Laparoscopic ISR may, therefore, be a useful technique for giant rectal GIST. The advantages of this procedure are: 1. it may preserve anal function for tumors located in a lower position of the rectum and 2. it may ensure a safe surgical margin by extending to the intersphincteric plane through the abdomen and in resection of distal margins under a direct view through the anus [9]. However, few reports have studied the benefits of laparoscopic ISR for rectal GIST. Fuimoto et al. performed laparoscopic ISR for 5 patients with a rectal GIST after preoperative chemotherapy. All cases achieved curative resection and recurrence did not occur for any patient during the 1- to 4-year postoperative period [10].

A review of cases of patients in Japan who had successful anal-preserving surgery following preoperative chemotherapy with IM is provided (Table 1). The median tumor size was 57.5 mm, and the median tumor response rate was 33.5%, with 21 patients exhibiting a PR (75%) and seven exhibiting SD (25%). The median period for chemotherapy with IM was 4 months, and laparoscopic ISR was performed in 10 of the cases (35.7%). The current case represented the largest tumor where treatment preserved the anus using laparoscopic surgery.

**Table 1:** A review of cases of patients who had successful anal-preserving surgery following preoperative chemotherapy with IM.

Case	Author	Year	Age	Size (mm)	Response Rate (%)	NAC (a) Period (month)	Surgical Procedure
1	Takahashi	2005	44	30	0	3	Transsacral resectioln
2	Natori	2007	30	50	50	4	Transsacral resectioln
3	Ebihara	2008	63	60	31.2	5	Transanal Resection
4	Sekoguchi	2009	64	63	31.2	4	Transanal Resection
5	Yamakawa	2011	62	28	14	11	Lap-ISR (b)
6	Sakon	2011	52	50	22	3	Transanal Resection
7	Matsuo	2011	75	25	56	6	Lap-ISR
8	Kato	2012	55	112	61.6	4	ISR
9	Nakajima	2012	58	66	68	6	Lap-ISR
10	Nakajima	2012	66	62	53	3	Lap-ISR
11	Nakamura	2012	56	80	37.5	3	Lap-ISR
12	Hirata	2013	94	80	38	4	Transsacral resectioln
13	Ide	2013	30s	55	32.7	11	Lap-ISR
14	Babaya	2014	60	24	0	1	Transperineal resection
15	Ishii	2014	43	90	50	6	Partial ISR
16	Yaguda	2014	77	42	45.2	5	Transperineal Resection
17	Shimomura	2015	63	60	33	6	Lap-ISR
18	Torashima	2015	59	79	41.7	7	ISR
19	Arimoto	2016	60	30	30	2	Lap-ISR
20	Takeda	2016	76	80	30	4	Lap-sLAR (c)
21	Tate	2016	43	45	15.5	1.5	Lap-sLAR
22	Kosuge	2016	65	30	50	3	Transsacral resectioln
23	Tazawa	2017	75	53	34	4	ISR
24	Yoshioka	2017	67	50	24	1	Transanal Resection
25	Fukuda	2017	76	28	61	6	Lap-ISR
26	Kenno	2017	40s	90	53	6.5	Transperitoneal Resection
27	Mizutani	2019	56	60	11	6	Transperineal resection
28	Our case	2019	55	100	30	9	Lap-ISR

The median tumor size was 57.5 mm, and the median tumor response rate was 33.5%, with 21 patients exhibiting a PR (75%) and seven exhibiting SD (25%). The median period for chemotherapy with IM was 4 months, and laparoscopic ISR was performed in 10 of the cases (35.7%).

a NAC: Neoadjuvant chemotherapy.

b Lap-ISR: Laparoscopic intersphincteric resection.

c sLAR: Super lower anterior resection.

## 4 Conclusion

Preoperative chemotherapy with IM for giant rectal GIST is effective because it can be expected to reduce tumor size, allowing laparoscopic ISR to be a safe surgical technique for the preservation of the anus when treating a large rectal GIST.

## Declaration of Competing Interest

The authors have no conflicts of interest to declare.

## Funding

This research did not receive any specific grant from funding agencies in the public, commercial, or not-for-profit sectors.

## Ethical approval

This case report was exempted from ethical approval from the institution.

## Consent

Written informed consent was obtained from the patients for publication of this case report and accompanying images.

## Author contribution

S.N and N.M wrote this manuscript. S.N, N.M, T.T, H.I, S.F, T.O, H.T, M.U, C.M, T.M, MM and Y.D had medical conference to decide this surgical procedure. All authors approved the manuscript to be published, and agree to be accountable for all aspects of the work in ensuring that questions related to the accuracy or integrity of any part of the work are appropriately investigated and resolved.

## Registration of research studies

This paper is a clinical report, so the authors declare that no registration is needed.

## Guarantor

Norikatsu Miyoshi is the Guarantor for this work.

## Provenance and peer review

Editorially reviewed, not externally peer-reviewed.
